# Gallstones in the Era of Metabolic Syndrome: Pathophysiology, Risk Prediction, and Management

**DOI:** 10.7759/cureus.80541

**Published:** 2025-03-13

**Authors:** Ke Wang, Zhigang Liu, Rongmei Tang, Yanguang Sha, Zhilin Wang, Yisheng Chen, Guangbin Chen

**Affiliations:** 1 Surgery, Wannan Medical College, Wuhu, CHN; 2 Hepatobiliary Surgery, The Second People's Hospital of Wuhu, Wuhu Hospital Affiliated to East China Normal University, Wuhu, CHN; 3 General Surgery, Wuhu Guangji Hospital, Wuhu, CHN

**Keywords:** dyslipidemia, gallstones, gut microbiota, insulin resistance, metabolic syndrome, obesity, pathophysiology, preventive strategies, risk prediction, therapeutic approaches

## Abstract

Gallstone disease (GSD) and metabolic syndrome (MetS) are increasingly prevalent conditions with significant global health implications. Recent evidence highlights a strong epidemiological association between these disorders, driven by shared pathophysiological mechanisms. This review provides a comprehensive analysis of the intricate relationship between MetS and GSD, focusing on the role of insulin resistance, dyslipidemia, obesity, and gut microbiota dysbiosis in gallstone formation. An integrated pathophysiological model is proposed, linking metabolic disturbances to bile cholesterol supersaturation, gallbladder dysmotility, and chronic inflammation. The review also explores clinical implications, including risk prediction models based on metabolic parameters, early detection biomarkers, and targeted interventions such as lifestyle modifications, pharmacological therapies, and microbiome modulation. By addressing the metabolic underpinnings of GSD, this synthesis offers a foundation for developing preventive and therapeutic strategies to mitigate the burden of these interconnected conditions. Future research directions are outlined to refine mechanistic insights and improve clinical outcomes.

## Introduction and background

Gallstone disease (GSD) represents one of the most common digestive system disorders worldwide, with increasing prevalence rates approaching 6% of the global population [1]. The geographic distribution shows significant variation, with traditionally lower rates in Asian countries now experiencing rapid upward trends. While often asymptomatic, GSD can manifest as chronic pain, episodic discomfort, nausea, upper abdominal colic, diarrhea, and anorexia [2]. More severe complications include acute cholangitis, acute cholecystitis, and biliary pancreatitis, which may necessitate cholecystectomy. Approximately 10% of asymptomatic patients develop symptoms within five years of diagnosis and 20% within 20 years [3].

Concurrently, metabolic syndrome (MetS) has emerged as a major public health concern, characterized by a constellation of interconnected physiological, biochemical, and metabolic factors including abdominal obesity, glucose metabolism abnormalities, insulin resistance, hypertension, and dyslipidemia [4]. The prevalence of MetS continues to rise globally, currently affecting 41.8% of the adult population [5].

The convergence of these two conditions presents a significant clinical and economic burden on healthcare systems worldwide. Despite advances in our understanding of GSD genetics and pathophysiology, current treatment approaches remain predominantly invasive and surgical [6]. Therefore, elucidating the relationship between GSD and MetS represents a critical step toward developing effective preventive strategies, particularly for high-risk populations.

This review aims to comprehensively examine the relationship between GSD and MetS through an integrated framework that bridges epidemiological associations with mechanistic explanations. By establishing this connection, we seek to provide a theoretical foundation for novel preventive and therapeutic approaches that address both conditions simultaneously.

## Review

Gallstones and MetS: epidemiological associations

Multiple large-scale epidemiological studies have consistently demonstrated a significant association between MetS and GSD. This relationship persists across diverse populations, suggesting fundamental biological connections rather than coincidental findings.

Prevalence Patterns and Risk Stratification

Cross-sectional studies involving thousands of participants have established that the prevalence of GSD in patients with MetS significantly exceeds that in the general population [7]. A comprehensive analysis of 5,297 participants revealed that an elevated body roundness index (BRI) - a more accurate predictor of GSD than traditional BMI - correlates with increased GSD risk [8]. This association showed variation across demographic subgroups, with stronger correlations observed in women and middle-aged to elderly individuals.

Component-Specific Associations

When examining individual components of MetS, glucose metabolism abnormalities emerge as particularly significant risk factors for GSD. The National Health and Nutrition Examination Survey demonstrated that each unit increase in the triglyceride-glucose (TyG) index corresponded to a 41% increased risk of GSD, suggesting its potential utility as a predictive marker [9].

Dyslipidemia also exhibits strong associations with GSD formation [10]. Hypertriglyceridemia and low high-density lipoprotein cholesterolemia correlate closely with GSD occurrence. However, recent evidence suggests a nuanced relationship, with GSD incidence increasing as triglyceride levels rise but declining when exceeding 2.57 mmol/L [11]. Interestingly, some studies indicate that lower serum total cholesterol (TC) may paradoxically increase GSD risk, highlighting the complex nature of lipid metabolism in gallstone pathogenesis [11,12].

While the relationship between hypertension and GSD appears less pronounced than other MetS components, certain studies report elevated GSD prevalence in hypertensive patients [13]. The potential mechanism may involve chronic low-grade inflammation and oxidative stress associated with hypertension, which could compromise gallbladder wall integrity, contractile function, and bile excretion [14].

Synergistic Effects and Dose-Response Relationship

Beyond individual components, evidence indicates a dose-response relationship between the number of MetS components present and GSD risk [15]. Patients exhibiting three or more MetS components face substantially higher GSD risk compared to those with fewer components, suggesting synergistic rather than merely additive effects [16,17].

Pathophysiological foundation: an interactive model

Understanding the relationship between GSD and MetS requires examining their common pathophysiological bases and establishing an integrated model that explains their interactions.

Gallstone Classification and Formation Process

GSD encompasses several subtypes classified based on composition: cholesterol stones (the most common globally), pigment stones, and mixed stones [18]. Cholesterol stone formation involves a complex sequence beginning with cholesterol supersaturation in bile, followed by precipitation of solid cholesterol monohydrate crystals [19].

The cholesterol in the human body originates from three primary sources: de novo synthesis via acetyl coenzyme A, enterohepatic circulation, and dietary intake [20]. Since humans lack enzymes capable of degrading the sterol ring structure, excess cholesterol must be metabolized into other compounds or excreted through feces [21].

The liver secretes cholesterol into bile, where it is solubilized by bile acid micelles and phospholipids. Excess cholesterol is carried by cholesterol-rich phospholipid-cholesterol vesicles, which have a propensity for aggregation due to surface charge distribution or hydrophobic interactions [22]. This aggregation leads to localized high cholesterol concentrations and the formation of cholesterol monohydrate crystals - the initial nucleation core of cholesterol stones [23].

Additionally, neutrophil extracellular traps play a role in this process. Concentrated granulocytes, particularly neutrophils, extrude their DNA, which attaches to different cholesterol crystals [24]. Over time, these DNA-wrapped crystals combine, eventually forming larger stones.

MetS: Unified Pathophysiology

MetS represents a complex interplay of metabolic dysregulations centered around insulin resistance as the common pathophysiological mechanism [25]. This insulin resistance impairs the effective regulation of glucose, lipids, and other metabolic processes, triggering cascading metabolic disturbances.

The most widely applied diagnostic criteria from the National Cholesterol Education Program Adult Treatment Panel III define MetS as the presence of three or more of the following: abdominal obesity, elevated triglycerides, reduced high-density lipoprotein cholesterol (HDL-C), hypertension, and elevated fasting blood glucose [26].

The Integrated Pathophysiological Model

We propose an integrated model where MetS and GSD interact through multiple bidirectional pathways, with insulin resistance serving as the central pathophysiological link. Insulin resistance directly impacts hepatic cholesterol synthesis and secretion, bile acid metabolism, and gallbladder motility and function. This central mechanism operates in concert with lipid metabolism disorders characteristic of MetS, which lead to altered bile composition with cholesterol supersaturation, disrupted phospholipid-bile acid ratios, and compromised gallbladder membrane function [27-29].

The pathogenic process is further amplified through chronic low-grade inflammation and oxidative stress present in MetS, which damage gallbladder epithelium, promote mucin secretion, and facilitate nucleation processes [30,31]. Additionally, MetS-associated gut microbiota dysregulation plays a crucial role by affecting bile acid transformation and reabsorption, modulating farnesoid X receptor (FXR) signaling, and influencing the gallbladder inflammatory environment [32]. This comprehensive model provides a framework for understanding how different MetS components synergistically contribute to GSD formation through interconnected mechanisms rather than isolated pathways.

**Figure 1:**
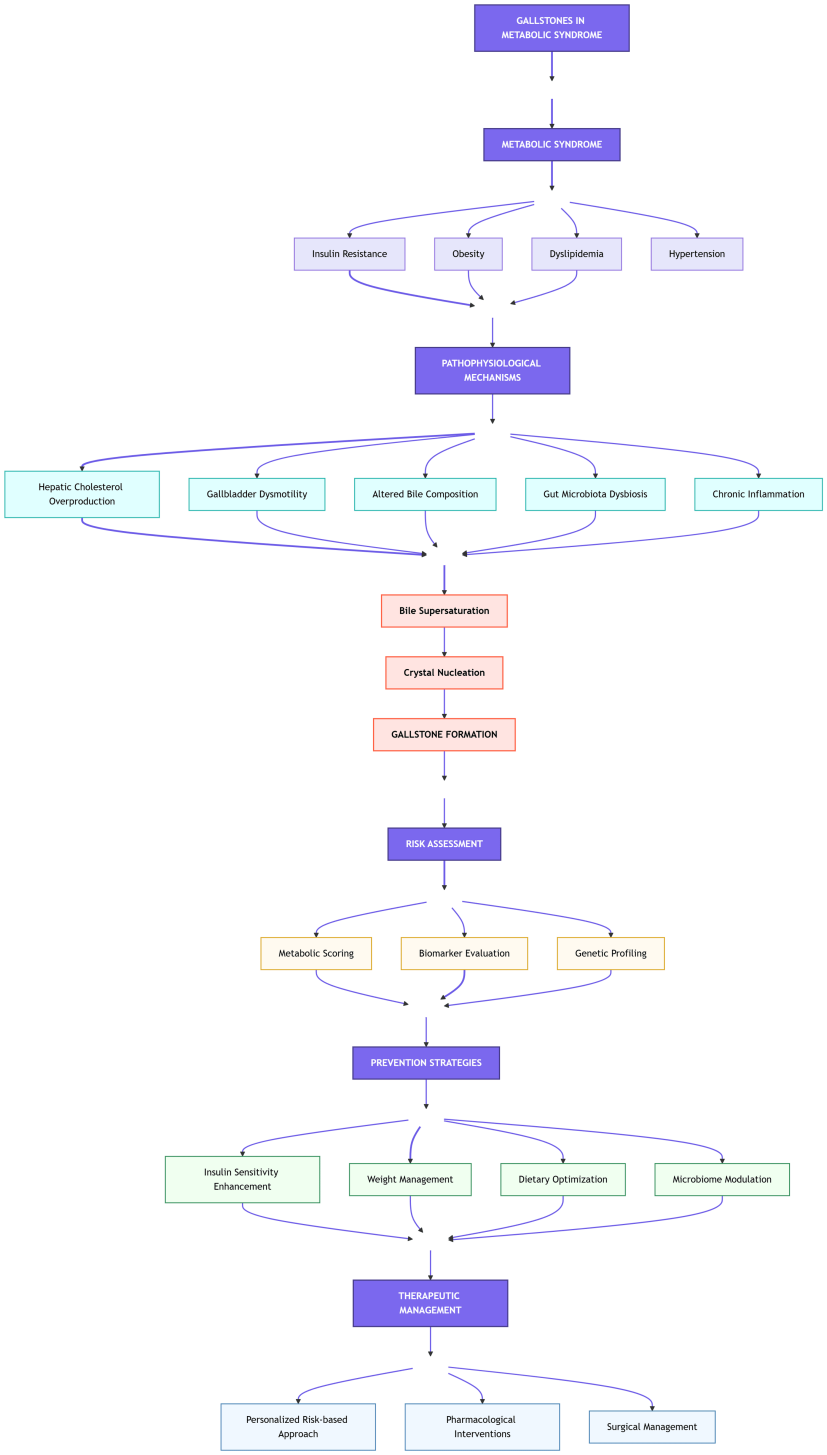
Pathophysiological mechanisms, risk assessment, and management strategies for GSD in MetS This flowchart illustrates the complex relationship between MetS and GSD through four major interconnected sections: (A) Core MetS components (insulin resistance, obesity, dyslipidemia, and hypertension) that initiate pathological processes; (B) Key pathophysiological mechanisms, including hepatic cholesterol overproduction, gallbladder dysmotility, altered bile composition, gut microbiota dysbiosis, and chronic inflammation, which lead to bile supersaturation and subsequent gallstone formation; (C) Risk assessment approaches incorporating metabolic scoring, biomarker evaluation, and genetic profiling; and (D) Evidence-based prevention and therapeutic strategies, focusing on insulin sensitivity enhancement, weight management, dietary optimization, and microbiome modulation. Arrows indicate the directional flow and relationships between different components. Color coding differentiates major pathways and hierarchical relationships: primary mechanisms (purple), pathophysiological processes (blue), risk assessment tools (orange), and intervention strategies (green). GSD, gallstone disease; MetS, metabolic syndrome Image credits: Guangbin Chen

Figure 1 presents a comprehensive vertical flowchart illustrating the intricate relationship between MetS and GSD. The diagram is structured in a hierarchical manner, beginning with MetS and its core components (insulin resistance, obesity, dyslipidemia, and hypertension) at the top. These components trigger various pathophysiological mechanisms, including hepatic cholesterol overproduction, gallbladder dysmotility, altered bile composition, gut microbiota dysbiosis, and chronic inflammation. These mechanisms converge to promote bile supersaturation, leading to crystal nucleation and ultimately gallstone formation. The flowchart then branches into evidence-based risk assessment tools, incorporating metabolic scoring, biomarker evaluation, and genetic profiling. Finally, it culminates in prevention strategies and therapeutic management approaches, emphasizing insulin sensitivity enhancement, weight management, dietary optimization, and microbiome modulation. The diagram employs a color-coded system to differentiate between primary pathways (purple), pathophysiological processes (blue), risk assessment tools (orange), and intervention strategies (green), facilitating clear visualization of the complex relationships between different components.

Mechanistic pathways linking MetS to gallstone formation

Insulin Resistance: The Central Mechanism

Insulin resistance represents the core pathophysiological mechanism connecting MetS to GSD formation. This connection operates through multiple pathways:

Enhanced cholesterol synthesis: Hyperinsulinemia stimulates 3-hydroxy-3-methylglutaryl-coenzyme A reductase, the rate-limiting enzyme for hepatic cholesterol synthesis [33]. Research by Dorvash et al. demonstrated that metformin, beyond lowering blood glucose, reduced GSD formation in C57BL/6 mice, suggesting that hyperinsulinemia accelerates hepatic cholesterol synthesis, increasing bile cholesterol saturation [34].

Altered receptor function: Hyperinsulinemia induces low-density lipoprotein receptor (LDL-R) production, enhancing their upregulation and activity. LDL binding to LDL-R promotes LDL entry from the blood into the liver, further increasing hepatic cholesterol synthesis and biliary cholesterol secretion [35].

Disrupted FXR signaling: Insulin resistance affects the FXR signaling pathway, which regulates cholesterol 7α-hydroxylase, the rate-limiting enzyme in bile acid synthesis [36]. FXR coordinates bile acid, lipid, and glucose balance through actions in hepatocytes and intestinal cells. In intestinal epithelial cells, FXR promotes fibroblast growth factor 19 (FGF19) gene expression [37]. After secretion and absorption, FGF19 reaches the liver, where it inhibits bile acid synthesis by reducing CYP7A1 abundance [38]. This disruption in insulin-sensitive individuals contributes to altered bile composition.

Impaired gallbladder motility: Insulin resistance reduces gallbladder sensitivity to cholecystokinin, impairing contractile function and promoting bile stasis - a critical factor in GSD formation [39].

Lipid Metabolism Disorders and Gallstone Formation

MetS-associated dyslipidemia contributes significantly to GSD pathogenesis through multiple mechanisms:

Cholesterol homeostasis disruption: MetS patients typically exhibit elevated triglycerides, increased LDL cholesterol, and decreased HDL-C. These alterations lead to increased cholesterol content in bile, promoting cholesterol supersaturation - a prerequisite for cholesterol crystal formation [40]. However, the relationship between serum cholesterol and GSD risk appears complex, with some Mendelian randomization studies suggesting that lower cholesterol levels might independently increase GSD risk [41].

Bile composition alterations: Dyslipidemia affects the crucial ratio of phospholipids and bile acids in bile. These components maintain cholesterol in a dissolved state, and their imbalance decreases cholesterol solubility [42]. MetS patients typically show relatively lower phospholipid and bile acid content in bile, creating favorable conditions for stone formation.

Lipoprotein profile changes: Research by Srivastava et al. confirmed that GSD patients have significantly elevated serum leptin, TC, lipoprotein A, triglycerides, and apolipoprotein B levels, with decreased apolipoprotein A-1 and HDL-C levels [43]. HDL-C appears to be an independent protective factor against GSD, with elevated serum HDL-C potentially reducing GSD risk [44]. Genetic studies by Kanoni et al. identified six new GSD susceptibility loci associated with blood lipids, offering potential targets for genetic-level GSD prevention [45].

Leptin resistance pathway: Elevated triglycerides inhibit leptin transfer and transport at the blood-brain barrier, decreasing neurological sensitivity to leptin and fostering leptin resistance [46]. Since leptin normally helps clear excess cholesterol and increase blood cholesterol excretion, this resistance promotes bile cholesterol supersaturation and subsequently induces cholesterol stone formation [47].

Obesity and Gallstone Formation

Obesity, particularly abdominal obesity, contributes to GSD formation through several mechanisms:

Increased hepatic free fatty acid flux: Expanded adipose tissue, especially visceral fat, releases more free fatty acids that enter the liver, stimulating hepatic cholesterol synthesis [48]. Additionally, obesity reduces liver insulin sensitivity, further promoting cholesterol synthesis and secretion [49].

Gallbladder dysmotility: Research demonstrates that obesity impairs gallbladder emptying and contractile function, leading to bile stasis that favors cholesterol crystal formation and stone growth [50].

Leptin-mediated effects: Leptin, primarily secreted by white adipocytes in a pulsatile manner with circadian rhythmicity, influences GSD formation through multiple mechanisms [51]. According to Lee et al.’s research [52], elevated serum leptin concentrations correlate with canine GSD. Leptin reduces gallbladder contractility through its effects on fibroblasts, increasing gallbladder volume and promoting bile stasis. It also interacts with receptors on the gallbladder wall, inducing inflammation, and can induce hyperinsulinemia that prompts the liver to produce cholesterol-saturated bile [53].

Weight loss paradox: While obesity increases GSD risk, rapid weight loss also paradoxically heightens this risk. During rapid weight reduction, bile nucleation protein levels change, increasing stone nucleation capacity. Rapid weight loss mobilizes cholesterol too quickly, causing cholesterol supersaturation and gallbladder contractile dysfunction [24,54]. Research suggests that during rapid weight loss, providing a moderately high-fat diet and ursodeoxycholic acid can mitigate GSD formation risk [55].

Gut Microbiota Dysbiosis and Gallstone Formation

Emerging evidence highlights the critical role of gut microbiota in GSD pathogenesis, especially in the context of MetS:

Bacterial proliferation and mucin production: MetS patients show increased susceptibility to infection, facilitating bacterial proliferation [56]. These bacteria produce phospholipase, which degrades lecithin into precipitating stearic acid, promoting nucleation. Simultaneously, bacterial presence stimulates gallbladder epithelium to secrete mucin - a matrix that facilitates stone formation [57].

Bile acid metabolism disruption: Different gut microbiota secrete diverse metabolic enzymes that mediate deconjugation, dehydroxylation, and epimerization of bile acids [58]. Bacteria-producing bile salt hydrolase (BSH) is abundant in the intestine and catalyzes the hydrolysis of conjugated bile acids to produce free bile acids [59]. These free bile acids, as signaling molecules, activate hepatic FXR, inhibiting cholesterol 7α-hydroxylase expression and altering bile composition [60].

FXR-FGF19 signaling axis: Intestinal FXR activation by free bile acids induces FGF19 secretion from intestinal epithelial cells [61]. FGF19 enters the circulation and binds to fibroblast growth factor receptor 4 and Klothoβ on hepatocyte surfaces, downregulating cholesterol 7α-hydroxylase gene expression and reducing bile acid synthesis [62]. In MetS, gut microbiota dysbiosis enhances BSH activity, leading to intestinal microecological imbalance and disrupting this regulatory axis.

Cyclic dysregulation: Under pathological conditions such as poor diet, gut microbiota dysbiosis leads to enhanced BSH activity, increasing intestinal free bile acids [63]. This initiates a negative feedback regulatory mechanism that inhibits bile acid synthesis until BSH substrates become insufficient, at which point positive bile acid synthesis mechanisms activate [64]. However, with persistent high intestinal BSH activity and irrational dietary structure, this cycle repeats, creating chronic dysregulation [65].

From mechanism to clinical application: risk prediction and early intervention

Understanding the mechanistic pathways connecting MetS to GSD allows for the development of evidence-based risk prediction models and targeted early interventions. This section provides specific clinical parameters and thresholds to guide practice decisions.

Risk Prediction Models

Based on established mechanisms, we propose a comprehensive risk assessment framework for GSD in MetS patients that integrates multiple complementary approaches. The metabolic risk scoring system incorporates several key MetS parameters, including the TyG index as a surrogate for insulin resistance, BRI for evaluating obesity, HDL-C/triglyceride ratio for dyslipidemia assessment, and blood pressure parameters, providing a systematic approach to risk stratification [66-69].

This framework is enhanced by the incorporation of mechanism-related biomarkers that show promise for early GSD risk prediction, such as serum leptin levels, lipoprotein profiles (with particular emphasis on the apoB/apoA1 ratio), FGF19 serum concentrations, and inflammatory markers associated with MetS [70,71]. Furthermore, the identification of GSD susceptibility loci associated with lipid metabolism has opened new avenues for genetic risk assessment, offering particular value for individuals with a family history of GSD or MetS, thereby enabling a more personalized approach to risk prediction and management.

Early Intervention Strategies

Based on pathophysiological understanding, we recommend specific, quantifiable interventions to disrupt the MetS-GSD pathway. Primary interventions focus on enhancing insulin sensitivity through structured physical activity and metformin therapy, coupled with controlled weight management strategies including structured gradual weight reduction [72-74]. For high-risk individuals undergoing active weight loss, prophylactic ursodeoxycholic acid administration is recommended to prevent gallstone formation [75].

Comprehensive dietary modifications constitute another crucial intervention component, emphasizing increased dietary magnesium intake, higher consumption of cooked vegetables, dried fruits, and fiber-rich foods, along with balanced fat intake during weight loss phases [76-78]. Additionally, gut microbiota modulation through prebiotic supplementation and probiotic interventions represents a promising therapeutic approach for addressing both MetS and GSD pathophysiology [79,80]. These interventions, when implemented systematically, provide a multifaceted approach to disrupting the MetS-GSD pathway and improving patient outcomes.

Therapeutic strategies and management

Integrated Management Approach

Effective management of patients with both MetS and GSD requires an integrated approach addressing both conditions simultaneously:

Comprehensive risk assessment: Patients with either condition should undergo screening for the other, given their established association. GSD patients should receive metabolic evaluation, including blood pressure, glucose, and lipid profiles. Conversely, MetS patients should undergo regular ultrasound screening for GSD.

Multidisciplinary team approach: Optimal management requires collaboration between gastroenterologists, endocrinologists, dietitians, and surgeons to develop comprehensive treatment plans addressing both conditions.

Patient education: Enhanced health education regarding the relationship between MetS and GSD empowers patients’ self-management awareness and capabilities. Education should emphasize healthy lifestyle maintenance, regular physical examinations, and early symptom recognition.

Specific Treatment Considerations

Surgical management: For symptomatic GSD, laparoscopic cholecystectomy remains the gold standard treatment. Due to high stone recurrence rates after gallbladder-preserving procedures and increased gallbladder cancer risk, gallbladder-preserving stone removal procedures are generally not recommended for benign gallbladder diseases.

Pharmacological interventions: For MetS management, medication selection should consider potential impacts on GSD risk. In selecting antihypertensive medications, angiotensin-converting enzyme inhibitors, angiotensin receptor blockers, and calcium channel blockers represent preferred options, while lipid-regulating medications, particularly statins, offer dual benefits through their primary lipid-lowering effects and additional anti-inflammatory properties that may help reduce GSD risk. For glycemic control, metformin emerges as a particularly advantageous choice, not only for its established role in improving insulin sensitivity but also for its potential protective effects against gallstone formation, thereby providing comprehensive benefits in the management of both MetS and GSD risk.

Ursodeoxycholic acid: This medication may benefit selected high-risk patients, particularly during rapid weight loss phases, by improving cholesterol solubility in bile.

Unresolved questions and research perspectives

Despite significant advances in understanding the MetS-GSD relationship, several critical questions remain unresolved, particularly regarding the quality and comprehensiveness of available evidence.

Research Limitations and Controversies

Causality versus association: While robust epidemiological associations exist between MetS and GSD, establishing definitive causal relationships requires further prospective studies and mechanistic validation. Current cross-sectional studies and retrospective analyses cannot fully exclude confounding factors or determine temporal sequences.

Human validation limitations: While animal models have provided compelling evidence for several key pathogenic mechanisms in GSD formation, significant gaps exist in translating these findings to human subjects. Although experimental studies strongly support the roles of insulin resistance, FXR-FGF19 signaling axis, and gut microbiome in GSD pathogenesis, direct measurements of their specific effects in humans remain limited, with most evidence derived from indirect measurements or observational studies rather than prospective interventional research. The causal relationships between these mechanisms and GSD development, particularly the efficacy of targeted interventions affecting bile acid metabolism and gallbladder function, require more comprehensive validation in human studies to bridge the gap between mechanistic insights from animal models and clinical applications.

Methodological limitations: Significant methodological limitations exist in current MetS-GSD research, primarily stemming from diagnostic and measurement inconsistencies. The reliance on self-reported medical histories and single-point ultrasound examinations introduces potential GSD diagnosis misclassification, while the lack of standardized measurement methods for MetS components across different studies compromises inter-study comparability. Furthermore, the predominance of cross-sectional and case-control study designs, coupled with limited longitudinal investigations, constrains our ability to establish definitive causal relationships between MetS and GSD, highlighting the need for more rigorous methodological approaches in future research.

Demographic variation understanding gaps: The current understanding of population-specific variations in the MetS-GSD relationship remains incomplete, particularly regarding geographic, ethnic, and gender differences that are crucial for developing targeted prevention strategies. This knowledge gap is especially pronounced in Asian populations, where despite rapidly rising GSD incidence, research on the MetS-GSD relationship remains relatively limited. Additionally, the lack of comprehensive data on age-specific risk profiles and intervention effectiveness across different life stages further hampers the development of population-tailored preventive approaches, emphasizing the need for more detailed demographic-specific research.

Emerging Research Directions

Novel therapeutic targets: Emerging understanding of the mechanisms linking MetS and GSD has revealed several promising therapeutic targets for intervention. The development of FXR agonists offers potential benefits in metabolic regulation and bile acid homeostasis, while targeted microbiome interventions focusing on bile acid-metabolizing bacteria represent an innovative approach to disease management. Additionally, novel strategies aimed at enhancing gallbladder motility in insulin-resistant states provide another avenue for therapeutic intervention, collectively suggesting multiple pathways for developing more effective treatments for patients with concurrent MetS and GSD.

Advanced imaging and biomarkers: The development of noninvasive methods for the early detection of gallbladder dysfunction and bile composition changes in MetS patients would facilitate earlier intervention.

Precision medicine approaches: Integrating genetic, metabolic, and microbiome profiles may enable personalized risk assessment and tailored preventive strategies for GSD in MetS patients.

Long-term outcomes research: Investigating whether addressing MetS components reduces long-term GSD complications would provide valuable evidence for preventive approaches.

## Conclusions

The relationship between MetS and GSD represents a complex bidirectional interaction underpinned by shared pathophysiological mechanisms, including insulin resistance, lipid metabolism disorders, obesity, and gut microbiota dysbiosis. Our comprehensive review establishes an integrated framework that bridges epidemiological observations with mechanistic explanations, providing a foundation for enhanced clinical management strategies.

Understanding these connections enables improved risk prediction and targeted interventions, where addressing MetS components through lifestyle modifications, appropriate pharmacological interventions, and potential microbiome modulation may simultaneously reduce GSD risk, ultimately improving patient outcomes. This integrated approach, supported by emerging mechanistic insights, offers promising directions for both the prevention and treatment of these increasingly prevalent conditions.
